# Research progress on circular RNA vaccines

**DOI:** 10.3389/fimmu.2022.1091797

**Published:** 2023-01-12

**Authors:** Yu Bai, Dong Liu, Qian He, Jianyang Liu, Qunying Mao, Zhenglun Liang

**Affiliations:** ^1^ Division of Hepatitis and Enterovirus Vaccines, National Institutes for Food and Drug Control, Beijing, China; ^2^ NHC Key Laboratory of Research on Quality and Standardization of Biotech Products, National Institutes for Food and Drug Control, Beijing, China; ^3^ NMPA Key Laboratory for Quality Research and Evaluation of Biological Products, Institute of Biological Products, National Institutes for Food and Drug Control, Beijing, China

**Keywords:** circular RNA vaccines, research progress, production process, quality control, outlook

## Abstract

Owing to the success of linear mRNA coronavirus disease 2019 (COVID-19) vaccines, biopharmaceutical companies and research teams worldwide have attempted to develop more stable circular RNA (circRNA) vaccines and have achieved some preliminary results. This review aims to summarize key findings and important progress made in circRNA research, the *in vivo* metabolism and biological functions of circRNAs, and research progress and production process of circRNA vaccines. Further, considerations regarding the quality control of circRNA vaccines are highlighted herein, and the main challenges and problem-solving strategies in circRNA vaccine development and quality control are outlined to provide a reference for circRNA vaccine-related research.

## 1 Introduction

Coronavirus disease 2019 (COVID-19) vaccines are currently the first vaccines to be rapidly and successfully developed and applied through multiple technological routes following the publication of a pathogenic sequence (1–4). These vaccines include inactivated vaccines, recombinant protein vaccines, adenovirus vector vaccine and mRNA-based vaccines, with the COVID-19 mRNA-based vaccines being the first-ever approved mRNA vaccines (5, 6). Currently, approximately 40 mRNA vaccines are undergoing clinical trials and 2 are commercially available. More than 4 billion doses of mRNA vaccines have been administered globally, accounting for approximately one-third of the total number of immunizations (7–9). mRNA vaccines not only induce high levels of humoral immunity, but also elicit relatively strong cellular immune responses (10–13). Owing to the rapidness and editability of the mRNA vaccine technology, it is suitable for use as a vaccine platform to tackle emerging infectious diseases (14, 15).

All of the aforementioned COVID-19 mRNA vaccines possess a linear structure. At present, modified linear mRNA molecules still have poor stability, which limits the amount of protein that can be expressed (16–19). Besides linear RNAs, circular RNAs (circRNAs) exist in nature. Such RNAs have a single-chain closed-loop circular structure formed by covalent bonds that results in greater stability *in vivo* compared with linear RNAs (20). circRNAs are also capable of protein encoding and translation, which confers potential for vaccine development (21). The successful development and application of COVID-19 mRNA vaccines have prompted researchers to explore the use of circRNAs for the development of higher-stability nucleic acid-based COVID-19 vaccines. Recently, several research teams successfully developed COVID-19 circRNA vaccines, which not only possess the advantages of linear mRNA vaccines, but also boast a greater stability at a range of temperatures and longer duration of protein expression than self-formulated linear mRNA vaccines (22–24). Accordingly, low doses of circRNA vaccines may be sufficient for eliciting strong immune responses (22, 23). Researchers have attempted to utilize the stability of circRNAs for the direct synthesis of chimeric antigen receptor (CAR) T cells through the introduction of CAR-expressing circRNA into T cells to avoid the tedious process of producing CAR-T cells through T cell isolation followed by engineering (25). Accordingly, some researchers believe that circRNA vaccines may serve as powerful tools for tackling future emerging major infectious diseases or other frequent viral diseases, such as hepatitis B virus (HBV), human herpes virus (HHV), Ebola virus (EBV), influenza A virus (IAV) and human papillomavirus (HPV), and may be developed into therapeutic vaccines for tumors (22–29). Biopharmaceutical companies and research teams worldwide have therefore diverted considerable attention and research efforts to the development of circRNA vaccines. In this review, we aimed to summarize the research progress and formulation process for circRNA vaccines and highlight the considerations and outstanding issues in the quality control of RNA vaccines to provide a reference for future circRNA vaccine-related research.

## 2 Discovery of circRNAs

In 1971, virus-like molecules that can invade plants and cause plant death were discovered by researchers in a study on potato spindle tuber disease and given the name “viroids.” Unlike viruses, viroids are single molecules that lack a protein coat (25, 30). Sanger et al. first described viroids as single-stranded covalently closed circRNA molecules in 1976 (31). In 1979, Hsu and Coca-Prados discovered the presence of circRNA molecules without free flanking ends in HeLa cells using electron microscopy; this was the first reported observation of circRNA molecules in eukaryotic cells (32). The invention of the polymerase chain reaction (PCR) technique in 1985 led to a surge in research interest in linear RNA. As a result, only little attention was paid to circRNAs and some researchers believed that circRNAs were merely byproducts of linear RNAs; this led to the stagnation of circRNA-related research. In the early 1990s, sporadic studies identified and characterized circRNAs generated from endogenous RNAs (25). The published reports indicated that the non-polyadenylated RNA with scrambled exons produced from non-canonical splicing (“scrambled exons”) are covalently closed circular RNAs, such as the specific circularization of *EST-1* gene transcripts (25, 33–36).

**Figure 1:**
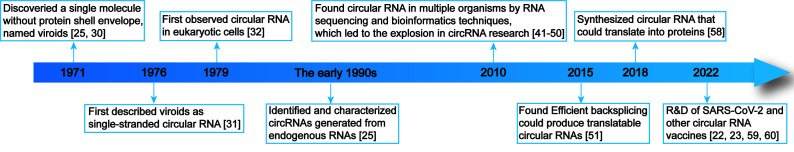
Key discoveries and advances in research on circRNAs.

In the following years, a few studies proposed mechanisms by which these molecules could be generated, such as the hypothesis that inverted repeats are necessary for *Sry* circularization (37–39). And additional circular RNAs could be produced from the rat cytochrome P450 2C24 gene, the human cytochrome P450 gene and others (37, 38). In 2006, researchers discovered the presence of circular transcripts in *Drosophila melanogaster (* 40). Beginning in about 2010, with the rapid development of RNA sequencing and bioinformatics techniques, studies involving the combined use of such techniques revealed the presence of diverse and highly conserved circRNAs in different organisms, such as humans, mice, plants, *Cryptococcus*, zebrafish, and protists, which led to an explosion in circRNA research (41–50). In 2015, circRNAs capable of protein encoding and translation were first reported (51). From 2017 onward, the extensive application of modern molecular biological experimental techniques to evaluate circRNAs enabled the validation of various circRNAs previously discovered through RNA sequencing and bioinformatics studies (52–57). In 2018, researchers successfully synthesized circRNAs capable of protein expression, which may be applied to vaccine development (58). In 2022, some research teams embarked on studies on COVID-19 circRNA vaccines and therapeutic vaccines for tumors or genetic diseases (**Figure 1**) (22, 23, 59, 60). With the continuous increase in the depth of circRNA research, circRNAs have been found to play important roles under certain physiological and pathological conditions, and are capable of protein expression, which might enable their incorporation into vaccines (61–64). Therefore, circRNAs have gradually become an area of great interest in molecular biology and other related fields of research (65, 66).

## 3 *In vivo* metabolism and functions of natural circRNAs

### 3.1 Synthesis of circRNAs

To date, RNAs have been recognized to be circularized *in vivo* by back-splicing and exon skipping, with back-splicing potentially being more prevalent owing to its seemingly more frequent observations than exon skipping (67). RNA circularization by back-splicing refers to the inverse ligation of the downstream 5’ splice donor site to the upstream 3’ splice acceptor site after the emergence of breakpoints in RNA. Thereafter, certain intron sequences undergo alternative splicing, which ultimately leads to the generation of circRNA (21). Two widely accepted models of RNA circularization exist, lariat-driven circularization and direct back-splicing circularization. Lariat-driven circularization involves exon skipping events and intron escaping from debranching while direct back-splicing circularization involves *Cis*-elements-mediated and *Trans*-factors-mediated events. RNA circularization by exon skipping refers to the process in which circularization occurs following the splicing of exons together with introns (67). The published studies revealed that different breakpoint locations contribute to the diversity of circRNAs, and different types of circRNAs are generated by the random combination of exons, introns, intergenic regions, or non-transcribed regions through back-splicing (41, 67).

**Figure 2:**
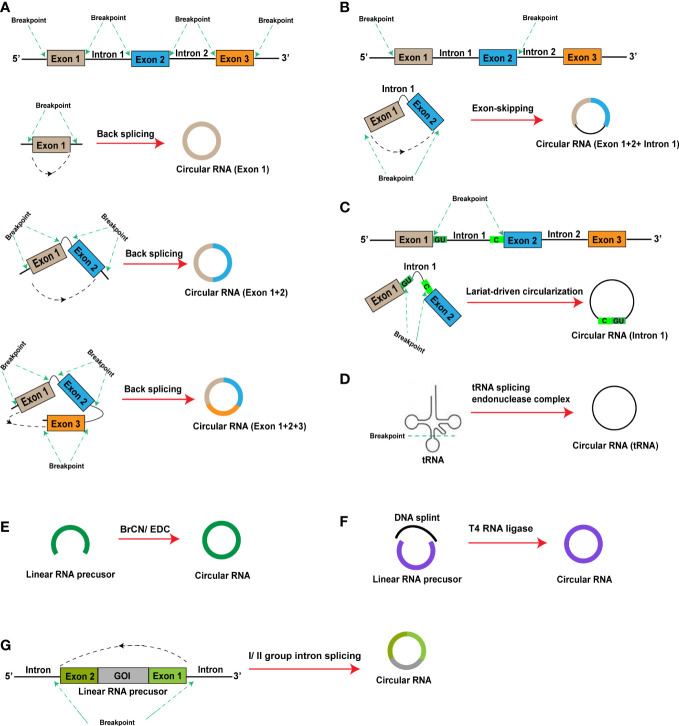
In vivo and in vitro (artificial) RNA circularization methods. **(A–D)** *In vivo* RNA circularization methods: **(A)** Exonic circRNA; **(B)** Exonic-intronic circRNA; **(C)** Intronic circRNA; **(D)** tRNA intronic circRNA; **(E, F)** *In vitro* (artificial) RNA circularization methods: **(E)** Chemical synthesis; **(F)** Ligation by T4 RNA ligase; **(G)** Intron self-splicing. BrCN, cyanogen bromide; EDC, 1-ethyl-3-(3-dimethylaminopropyl) carbodiimide; GOI, gene of interest.

**Figure 3:**
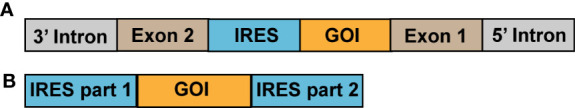
Methods currently used to formulate circRNA vaccines. **(A)** Circularization by intron splicing; **(B)** Ligation by T4 RNA ligase. GOI, gene of interest.

CircRNAs are generally classified into four main types based on differences in sequence combination: (1) Exonic circRNAs contain only exons, account for more than 80% of all identified circRNAs, and can be generated by lariat-driven circularization or direct back-splicing circularization. Base-pairing between the two flanking introns, which contain reverse complementary sequences, brings together a downstream 5′ ss and an upstream 3′ ss and facilitates back-splicing reactions to produce circular RNAs. (**Figure 2A**); (2) Exonic-intronic circRNAs, which are generated by exon-skipping circularization, harbor flanking intron sequences at the offside of core exons that should generally be spliced; therefore, exonic-intronic circRNAs are referred to as eiciRNAs or retained-intron circRNAs (**Figure 2B**); (3) Intronic circRNAs are also produced by a the intron escaping from debranching of lariat-derived mechanism relying on a consensus guanine (G)- and uracil (U)-rich domain near the 5′ splice site of pre-mRNA and a cytosine (C)-rich domain near the breakpoint. Intriguingly, the GU-rich domain can protect the C-rich domain from branching or degrading, thus generating stable circRNAs or so-called intron-derived circRNAs (**Figure 2C**); and (4) tRNA intronic circRNAs. For tRNA intronic circRNAs, pre-tRNAs are cleaved by the tRNA splicing endonuclease (TSEN) complex, and the exons and introns resulting from cleavage are ligated to form tRNA intronic circRNAs (**Figure 2D**) (41, 67–69).

According to the published studies, factors, such as sequence characteristics, protein regulators, and transcription stage, can affect the efficiency of back-splicing circularization (25, 41, 67). Indeed, beyond cis-elements, a number of RNA-binding proteins (RBPs) were found to actively modulate circular RNA biogenesis. Longer flanking intronic elements harboring several complementary or repeat sequences may contribute to higher circularization efficiency (25). Different splicing-associated proteins also exert different effects on circularization efficiency. For instance, adenosine deaminase acting on RNA-1 (ADAR1) suppresses RNA circularization (70).

### 3.2 Biological functions of circRNAs

According to several studies, circRNAs exist in various types of tissues in different species and possess multiple biological functions, such as the regulation of gene transcription and protein encoding (69, 71–74). CircRNAs also play important roles in the regulation of physiology and the occurrence, progression, and outcomes of a diverse range of diseases (75, 76).

#### 3.2.1 Regulation of gene transcription

Researchers have reported that circRNAs can bind to the DNA of host genes to form DNA: RNA hybrids known as R-loops, which affect DNA replication, transcription, and post-damage repair. For instance, *circSEP3* (derived from exon 6 of *SEPALLATA3*) causes transcriptional termination through R-loop formation with *cSMARCA5* (derived from exon 15 of *SMARCA5*) *(* 77). *circRHOT1* recruits Tat-interactive protein 60 kDa (TIP60) to the nuclear receptor subfamily 2 group F member 6 (*NR2F6*) promoter, thereby promoting cell proliferation, migration, and invasion in hepatocellular carcinoma through the induction of proto-oncogene expression (78).

#### 3.2.2 “Sponging” effect

Natural circRNAs exhibit a “sponging” effect toward microRNAs (miRNAs). For instance, *circHIPK3* (derived from exon 2 of homeodomain interacting protein kinase 3 (*HIPK3*)) can bind to *miR-124* and inhibit its activity, thereby preventing *miR-124* from inhibiting human cell proliferation (79); and *circHIPK2* regulates astrocyte activation by targeting *miR124-2HG (* 80). Studies have indicated that circRNAs may exhibit a sponging effect toward proteins. Y-box binding protein-1 (Ybx1) is a transcription factor of many genes and its significant overexpression is associated with poor outcomes and the recurrence of various common tumors. According to researchers, *circNfix* can bind to Ybx1 and the E3 ubiquitin ligase, Nedd4l, which promotes their interactions and induces Ybx1 degradation (81).

#### 3.2.3 Protein activation through complex formation

Many types of molecules in the host organism participate in the biological processes of host-virus interactions. A previous study revealed that the Epstein-Barr virus (EBV) produces the circRNA, *circBART2.2*, after infecting host cells, which can be recognized by the RIG-I receptor of the cells (82). Thereafter, RIG-I receptor activation occurs, enabling antiviral effects through subsequent activation of the downstream interferon signaling pathway. Another study found that circRNAs were not recognized by the RIG-I receptor after N6-methyladenosine (m6A) methylation, which serves as one of the mechanisms by which viruses evade host immune responses (83–85).

#### 3.2.4 Protein translation

In 2015, the ability of circRNAs to encode and translate proteins was first discovered in the fruit fly. This key finding provided a theoretical basis for the subsequent development of circRNA vaccines (51, 67). Several studies have reported that circRNAs translate their encoding proteins *via* internal ribosomal entry site (IRES)-mediated or m6A-induced ribosome engagement site (MIRES)-mediated translation initiation instead of 5’ cap-dependent translation initiation (41, 67, 84). IRESs are RNA elements that recruit ribosomes to an internal region of mRNA for the initiation of translation, and can promote ribosome assembly and initiate translation through the recruitment of different trans-acting factors (21). IRESs are utilized for translation by many types of circRNAs, including *circMbl* and *circFGFR1 (* 81). MIRES-mediated translation initiation involves the occurrence of m6A methylation at one or more sites in RNA, which enables the recruitment of eIF4G2 for the initiation of translation. For instance, *circE7*, which is encoded by human papillomaviruses (HPVs) and localized to the cytoplasm, contains m6A modifications and performs E7 oncoprotein translation (86). Another study reported that m6A modifications enhance IRES-mediated *circZNF609* translation efficiency (87).

### 3.3 CircRNA degradation

The stable closed-loop structure of circRNAs protects them from cleavage by exoribonucleases (67). Therefore, circRNAs are less easily degraded *via* routine RNA degradation pathways and are resistant to cleavage by the exoribonuclease RNase R in the short term (88). It is more stable than linear RNA, which is the natural advantage of circRNA vaccine development. Nevertheless, circRNAs are still degradable by certain means, including endonucleases. In a previous study, double-stranded RNA (dsRNA) or polyinosinic: polycytidylic acid (poly (I:C)) was found to activate the endonuclease, RNase L, *in vivo*, causing the global degradation of circRNAs (69). The ATP-dependent RNA helicase, upstream frameshift 1 (UPF1), and the endonuclease, G3BP1, can identify circRNAs with complex secondary structures and induce their degradation (67). circRNAs may undergo m6A modification, which is recognized by the m6A reader protein, HRSP12. Consequently, HRSP12 may interact with the RNase P/MRP endonuclease complex to induce circRNA degradation (89). Notably, circRNAs can be degraded by the endonuclease, RNase H, through targeting by specific primers and probes. The complementary binding of *miR-671* to the circRNA CDR1as sequence also induces AGO2-mediated circRNA degradation (52).

## 4 Research progress on circRNA vaccines

Prior to 2015, researchers had only observed the presence of non-protein-encoding circRNAs in organisms. The discovery of circRNAs capable of protein encoding and translation in the fruit fly in 2015 provided a theoretical basis for research and development of circRNA vaccines. With the continuous increase in the depth of circRNA-related research, three methods for the artificial preparation of circRNAs have emerged: (1) Chemical synthesis: cyanogen bromide (BrCN) or 1-ethyl-3-(3-dimethylaminopropyl) carbodiimide (EDC) induces covalent bond formation between the 5’-terminal phosphate and 3’-terminal hydroxyl groups of linear RNAs to produce circRNAs. The occurrence of side reactions, such as the formation of 2’-5’ phosphodiester bonds during the circularization reaction, is a limitation of this approach. Further, chemical bonds in RNA oligomers are usually less effective than those in DNA analogs (**Figure 2E**); (2) Ligation by T4 RNA ligase: a linear RNA molecule can be circularized through the covalent bonding of a 5’-terminal monophosphate to a 3’-terminal hydroxyl group under the effects of T4 RNA ligase. However, the circularization reaction can only occur under conditions of a single phosphate at the 5’ terminus. As RNA synthesized *in vitro* using T7 RNA polymerase contains a 5’-triphosphate terminus, two phosphate groups must be removed before circularization of the synthesized linear RNA. Presently, RNA 5’ pyrophosphohydrolase (RppH) is used for the direct removal of the β and γ phosphates (**Figure 2F**); (3) Intron self-splicing: group I and II introns can perform RNase functions, enabling self-splicing of linear RNA molecules to form circRNAs without assistance from other enzymes (**Figure 2G**).

In 2015, researchers constructed a single exon minigene containing split GFP, and found that the pre-mRNA indeed produces translatable circRNA through efficient back-splicing in human and Drosophila cells (51). In 2018, researchers synthesized circRNAs encoding the enhanced green fluorescent protein (EGFP) using the group I intron splicing circularization method with IRES-mediated translation initiation. This synthesis led to the successful expression of EGFP in 293T cells. Surprisingly, the circRNAs could perform continuous expression for up to 168 h, whereas expression by self-formulated mRNAs only lasted for 48 h. Currently reported artificially synthesized protein-expressing circRNAs have mainly been produced through intron splicing or ligation by T4 RNA ligase, with the constituent elements being different for the two circularization methods (**Figure 3**) (58).

Wei et al. recently developed a circRNA vaccine against the original COVID-19 strain by adopting the group I intron splicing RNA circularization method with IRES-mediated translation initiation, and using lipid nanoparticles (LNPs) as delivery systems. In mice separately immunized with doses of 10 μg/animal and 50 μg/animal, the serum neutralizing antibody titers at 5 weeks post-boost were approximately 10^3^ and 10^5^, respectively, with relatively strong Th1 immune responses elicited by both doses. When a challenge experiment using the original viral strain was performed after monkeys had received two doses of the vaccine at a dose of 100 μg/animal, the immunization group was found to have a significant decrease in viral load in the lungs, degree of lung injury, and number of infiltrating inflammatory cells in the lungs. The technology platform was subsequently utilized to generate a circRNA vaccine against the COVID-19 Delta variant, which was used for the immunization of mice at a dose of 10 μg/animal. At 7 weeks post-boost, the serum neutralizing antibody titer was approximately 10^4^ against the Delta variant, and reached approximately 10^3.5^ with the Omicron BA1 variant, thereby demonstrating the broad-spectrum activity of the vaccine. The formulated circRNA vaccine also had a greater stability than linear mRNA vaccines at 4°C, 25°C, and 37°C. Another COVID-19 circRNA vaccine prepared using the T4 RNA ligase method also displayed a certain level of immunogenicity in mice (22).

Seephetdee et al. adopted the group I intron splicing method for RNA circularization to formulate a COVID-19 circRNA vaccine using the spike (S) protein of the original COVID-19 strain as the antigen-binding construct and including multiple mutations (including K417N, L452R, T478K, E484K, N501Y, and D614G). Mice administered two immunizations of the vaccine at a dose of 5 μg/animal exhibited certain neutralizing activity against the Alpha, Beta, Delta, and Omicron variants and pseudoviruses. IFN-γ responses were also elicited in T cells (23).

Wang et al. formulated a COVID-19 circRNA vaccine using the group II intron splicing method for RNA circularization. When used for mice immunization, the vaccine elicited strong RBD-specific memory B cell responses and balanced Th1/Th2 cellular immune responses. The serum IgG titer of mice immunized with the circRNA vaccine was 10-fold that of mice immunized with a linear mRNA vaccine. Further, the circRNA vaccine exhibited good neutralizing activity against pseudo-SARS-CoV-2 and effectively blocked the binding of three types of receptor-binding domain (RBD) mutants (wild type, Delta, Omicron) to the hACE2 receptor on 293T cells. The COVID-19 circRNA vaccine could perform continuous expression in cells for 6 days, while expression by the self-formulated mRNA vaccine only lasted for 2 days (24).

The use of the T4 dt intron splicing method for RNA circularization causes the generation of “splicing scar” sequences, with different sequence lengths leading to different topological structures. A study revealed that differences in the constituent elements and compositions of circRNAs affected the translation efficiency, with vector topological structure, untranslated region (UTR), and IRES playing the most critical roles (26, 59). Through the experimental comparison of different splicing scar, 5’UTR, 3’UTR, and IRES sequences, the circRNA constructed using a 50 nt-long splicing scar sequence and the eIF4G-recruiting aptamer Apt-eIF4G sequence as the 5’UTR, full-length HBA1 sequence as the 3’UTR, and wild-type iCVB3 sequence as the IRES exhibited a considerable increase in translation efficiency and could perform continuous protein expression for up to 7 days in 293T cells. In contrast, the linear mRNA vaccine only achieved 3 days of continuous protein expression, with lower expression than that of the circRNA vaccine. The studies described above demonstrate that circRNA vaccines not only possess stronger immunogenicity, but also express proteins for a longer duration than self-formulated linear mRNA vaccines (90). Accordingly, stronger immune responses may be elicited by circRNA vaccines when administered at lower doses (22, 90).

The circRNA vaccines translated into proteins *via* IRES-mediated translation pathway. Similar to the linear mRNA vaccines, the circRNA vaccines are also translated into proteins in bodies to induce the robust humoral and effective cellular immunity, among which liposome and others could play the role in vaccine adjuvants. Some studies indicated that circRNA vaccines could continue to translate into proteins longer than the linear mRNA vaccines and even up to about 7 days (22, 58, 90). The mechanism might be that closed characteristics of circular RNAs theoretically prevent them from degradation by exonucleases that typically degrade linear RNAs from either 5′ or 3′ end (69). However, there is no research on how the circRNA vaccines degrades in bodies, and it may also be degraded *via* microRNA-mediated, m6A-mediated, RNase L-mediated, or other degradation pathways of biogenesis circRNAs.

Compare with peptide vaccines, inactivated virus vaccines and recombinant protein vaccines, circRNA vaccines could effectively induced the T cell immunity (22, 23). DNA vaccines have to come into the nucleus to be transcribed in mRNA, which leads to some risk for our bodies, whereas circRNA vaccines do not (1, 8). The published studies indicated that circRNA vaccines could continue to translate into proteins for a longer time compared with linear mRNA vaccines, suggesting that it has the better stability (24, 26, 90).

There are some obviously different between the circRNA and the linear mRNA vaccines. For example, the circRNAs and the linear RNAs have different degradation approaches *in vivo (* 25, 67). In additional, the circRNA vaccines mainly uses the IRES mediated-translation pathway, while linear mRNA vaccines use the 5’cap (m7GpppN) structure mediated-translation pathway. Thus, the circRNA and the linear mRNA vaccines translate into proteins *via* different transcription initiation complexes involving in different initiation factors (91–94). The obvious difference is that the circRNA vaccines do not require any modification because the annular structure is not easily degraded by exoribonuclease, whereas the linear mRNA vaccines need the pseudouridine modification, the 5’cap structure and the 3’poly(A) structure (7, 8, 22, 24).

## 5 Overview of the circRNA vaccine production process

**Figure 4:**
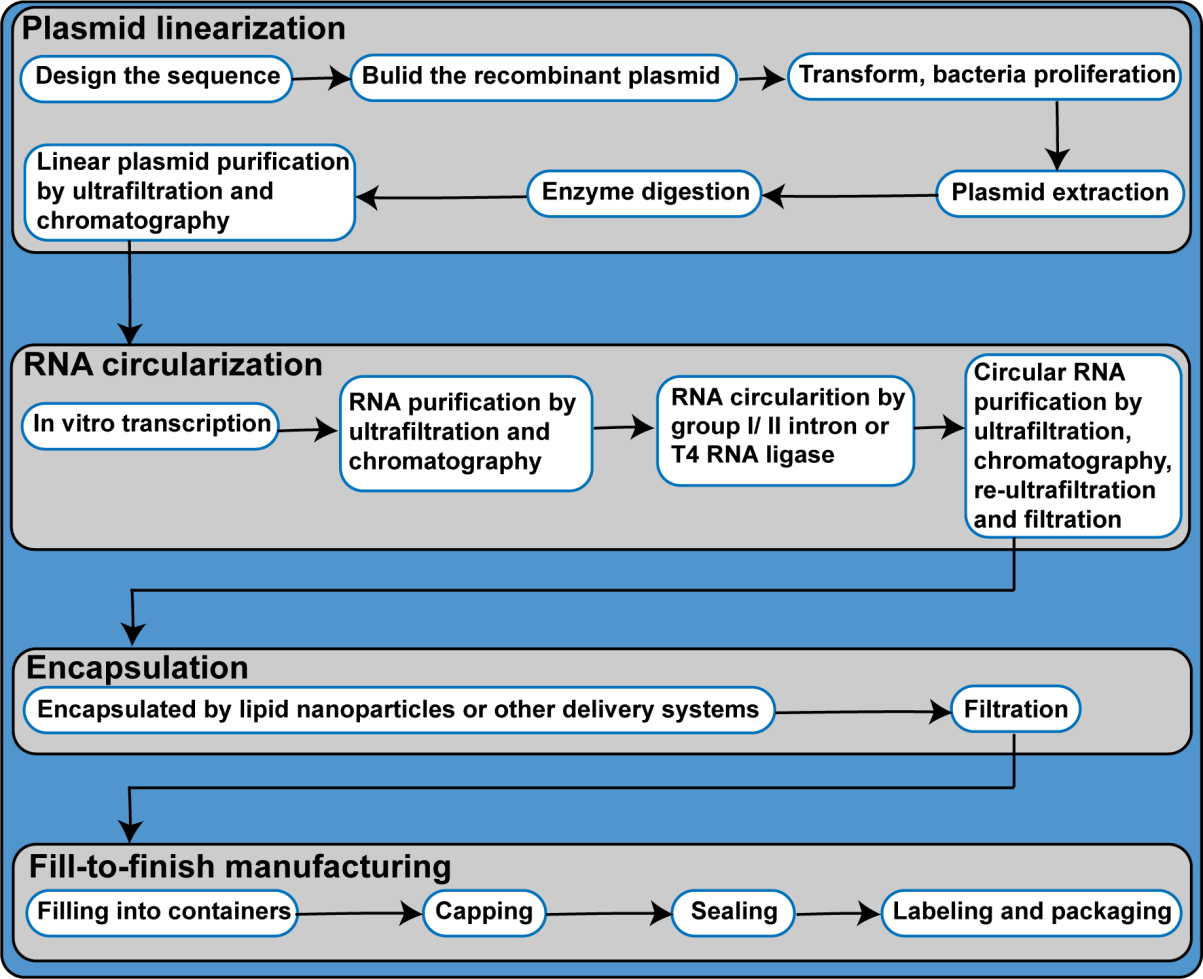
Process flow diagram of circRNA vaccine productions.

At present, the industrial production of circRNA vaccines is not mature, and most of them are in the stage of small-scale research. Reference can be made to the linear mRNA vaccine production process. The main differences are the cyclization process and the removal of associated impurities. Industrialized production of circRNA vaccines may be realized by the following process: (1) Synthesis of linear RNA molecules through *in vitro* transcription using plasmids as templates; (2) RNA circularization; and (3) Vaccine encapsulation using delivery systems, such as LNPs. Therefore, the acquisition of high-quality plasmids is of primary importance (90). The industrialized production process of plasmids is currently at a near-mature stage and consists of steps, such as fermentation, bacteria harvesting, lysis, clarification, ultrafiltration and concentration, and chromatographic purification. During the chromatographic purification step, the addition of different types of packing materials enables the removal of RNA, trace impurities, and endotoxins to obtain high-purity supercoiled plasmids. The obtained plasmids are subsequently subjected to single restriction endonuclease cleavage for linearization and chromatography for enzyme and salt removal. Linear RNA molecules are synthesized *via in vitro* transcription. Subsequently, the DNA template in the reaction products is removed using DNase I and the RNA product is purified by ultrafiltration. The linear RNA molecules are then subjected to circularization, which is a critical step in the circRNA vaccine preparation process. Currently, the intron splicing method holds the greatest promise for use as the circularization method in industrialized circRNA vaccine production as the addition of enzymes is not required. Ligation by T4 RNA ligase can also be used in industrialized circRNA production; however, its RNA circularization efficiency is significantly lowered during the circularization of RNA molecules with sequence length > 2000 bp. Furthermore, additional processes are required for the removal of T4 RNA ligase. By comprehensively considering various factors, ligation by T4 RNA ligase is identified to be less suitable for RNA circularization in industrialized production than intron splicing. Products of the circularization reaction include impurities, such as linear RNA precursors and spliced RNA sequences. The removal of linear RNA precursor molecules that had not been successfully circularized is a difficult problem faced by circRNA vaccine production due to the similarity of molecular weights between linear RNA precursors and the corresponding circRNA molecules. Researchers have reported the use of chromatography for the removal of linear RNA precursor molecules and other impurities from the circularization products (22). In the laboratory-scale production stage, high-performance liquid chromatography (HPLC) or RNase R digestion may be attempted for the removal of linear RNA precursors and other impurities (22, 24). However, RNase R is currently not suited for use in large-scale production due to its high cost. As the enhancement of RNA circularization efficiency leads to a reduction in impurity content, increasing circularization efficiency may be a feasible approach for the simplification of the purification process. A previous study demonstrated that RNA circularization efficiency could be effectively enhanced during the performance of circularization in a buffer solution with final concentrations of 50 mM Tris-HCl, 10 mM MgCl_2_, and 1 mM DTT at a pH of 7.5 (58). Finally, the circRNAs are encapsulated in delivery systems, such as LNPs, and subsequently subjected to filling and capping to form the final vaccine product (**Figure 4**). Of note, the circRNA vaccine formulation process does not require the addition of a 5’ cap and 3’ poly(A) tail or the removal of enzymes from reaction products (22).

## 6 Considerations for the quality control of circRNA vaccines

Presently, two COVID-19 linear mRNA vaccines have already been marketed; however, the quality control processes for these vaccines are relatively mature. The World Health Organization (WHO) issued a guidance document on regulatory considerations regarding the evaluation of the quality, safety, and efficacy of messenger RNA vaccines for the prevention of infectious diseases (95–97). Further, the U.S. Pharmacopeial Convention (USP) developed the Analytical Procedures for mRNA Vaccine Quality, and the Center for Drug Evaluation (CDE) of the China National Medical Products Administration (NMPA) published the Guiding Principles on Pharmaceutical Research Techniques for Prophylactic COVID-19 mRNA Vaccines (trial) (98, 99). Considering the characteristics of circRNA vaccines, existing guidance documents for mRNA vaccines, other types of vaccines, and nucleic acid therapeutics may serve as reference for the formulation of circRNA vaccine quality control processes (**Supplementary Material Table 1**) (22, 24, 90, 96–103).

CircRNAs are dependent on IRES or m6A modifications for translation initiation. Owing to their unique closed-loop structures, circRNAs are resistant to degradation *via* routine pathways, which eliminates the need for 5’ cap and 3’ poly(A) tail structures in the RNA molecules used to formulate circRNA vaccines (22, 23). Therefore, 5’ cap- and 3’ poly(A) tail-related quality control is not required for circRNA vaccines.

Quality control items emerging from core quality attributes (CQAs) related to the unique characteristics of circRNA vaccines must be emphasized. Circularization rate is the most important CQA of circRNA vaccines and has been measured by capillary electrophoresis (CE) and high-performance liquid chromatography (HPLC) in previous studies (22, 24). The accuracy of circRNA sequences should also be measured based on sequencing methods. However, the process for circRNA vaccines differs from that of linear mRNA vaccines as circRNAs require endonuclease cleavage for loop opening prior to sequencing (22, 24). During the RNA circularization process, single-stranded RNA (intronic sequences) may become detached and a portion of the linear RNA precursors ultimately remain uncircularized. Researchers have reported the use of CE and HPLC for determining circRNA vaccine purity (22). If ligation by T4 RNA ligase is adopted for the preparation of circRNAs, quality control of the residual T4 RNA ligase content of the vaccine product must also be performed, which can be measured by the enzyme-linked immunosorbent assay (ELISA). When RNase R is used for the removal of linear RNA precursor molecules, quality control is also required for residual RNase R content, which can also be measured by ELISA. Currently, specific antigen-antibody binding methods, such as ELISA, are adopted to measure the residual double-stranded RNA (dsRNA) content of mRNA vaccines, with the anti-dsRNA J2 antibody being mainly used (104, 105). However, studies that aim to determine whether circRNAs can directly bind the anti-dsRNA J2 antibody or interfere with the binding of anti-dsRNA J2 antibody to dsRNA have not been reported. Therefore, sample applicability studies must be performed before the adoption of ELISA to measure the residual dsRNA content of circRNA vaccines (104–106).

## 7 Outlook of circRNA vaccines

CircRNA vaccines may serve as a powerful tool for tackling future emerging major infectious diseases or frequent viral diseases and may be developed into therapeutic vaccines for tumors. Biopharmaceutical companies and research teams worldwide have therefore diverted considerable attention and research efforts to the development of circRNA vaccines. Currently, research on circRNAs remains in the preclinical stage due to various issues in research and development, production, quality control, and safety. Recently, researchers proposed that a blueprint for quality by digital design (QbDD) to support rapid RNA vaccine process development, manufacturing and supply (107). Accordingly, the QbDD concept should be incorporated into circRNA vaccine development, production, and quality control (108). The development and validation of new circRNA vaccine-related methods should be based on specific requirements stipulated in the General Chapter <1220> “The Analytical Procedure Lifecycle” recently released by the USP and the ICH Q2 “Validation of Analytical Procedures” and Q14 “Analytical Procedure Development” draft guidelines published by the International Council for Harmonization of Technical Requirements for Pharmaceuticals for Human Use (ICH), for which public comment is currently being sought. Further, an analytical target profile (ATP), risk analysis and identification, confirmation of CQAs, experimental design, process control, and in-use monitoring are required (108–113).

Future directions for the various aspects of circRNA vaccines are as follows:

Research and development: linear mRNA vaccines exhibit a “self-adjuvant” effect (4, 16, 106). The excess or deficiency of such effect is unfavorable for the induction of high levels of humoral immunity and effective cellular immunity. CircRNAs can activate the RIG-I and PKR cellular signal transduction pathways, which also provides a self-adjuvant effect (82, 88, 114–116). Therefore, the design and optimization of circRNA vaccines to activate the self-adjuvant effect to an appropriate degree are essential for enhancing vaccine efficacy. RNA circularization is a key stage in the circRNA vaccine formulation process, with circularization efficiency being a major determinant of vaccine production capacity, and high circularization efficiency being beneficial to the subsequent purification process (117). The design and optimization of the constituent elements and compositions of circRNA vaccines or development of novel RNA circularization methods are effective approaches for enhancing circularization efficiency. According to the published research, excessive RNA sequence length affects circularization efficiency. Therefore, during the design and optimization of target fragment length, care must be taken to avoid affecting the immunogenicity of the target protein or RNA circularization efficiency (90). CircRNA vaccines differ from mRNA vaccines as they possess a loop structure, and different target sequences may exhibit different conformations. Such characteristics of circRNAs may affect the RNA circularization efficiency and require adequate exploration during the research and development stage. Intron sequences play a key role in the RNA circularization reaction. Therefore, the optimization of intron sequences is a necessary step in the vaccine development stage (90). Exon length affects the efficiency of circularization by group I intron self-splicing, which also necessitates the optimization of exon sequence length during vaccine development. The selection of effective IRES structural elements is of great importance as it is a major determinant of protein translation by circRNA vaccines (24, 51, 58). Recently, it was reported that DeepCIP, the world’s first CircRNA IRES prediction tool, could predict the IRES that are more suitable for CircRNAs, so that CircRNAs can be adapted to different scenarios, such as vaccines and antitumor therapy (118). CircRNAs require delivery systems for encapsulation to form vaccines. LNPs currently serve as the main delivery system for circRNAs. The development of targeted LNPs may potentially contribute to the enhancement of vaccine protection effects. Alternatively, research efforts can be devoted to the development of novel and more effective delivery systems that can replace LNPs. For instance, some earlier studies have explored the use of exosomes and ferritin as delivery systems (119, 120).

Production: Attempts can be made to optimize the reaction conditions to achieve enhanced circularization efficiency. Under laboratory conditions, a re-execution of the circularization step with circularized RNA contributes to higher circularization efficiencies. Therefore, the development of a recircularization process and the incorporation of such process into vaccine production can be attempted (58). The similarity in molecular weight between circRNAs and the corresponding linear RNA precursor molecules poses difficulties for the elimination of linear RNA precursors from the vaccine product during the purification process. Currently, chromatography is mainly adopted for purification; however, there is a lack of reports on whether its purification effects are sufficient to meet production needs.

Quality control: As mentioned earlier, the molecular weights of circRNAs are similar to those of the corresponding linear RNA precursor molecules. Consequently, the two types of RNA molecules exhibit close peak appearance times during CE or HPLC to determine the RNA circularization rate. Accordingly, it is difficult to effectively distinguish between the two (22, 24). Further optimization of experimental conditions should be performed to satisfy the requirements for measurement. National and regional reference standards for dsRNA may also be needed in the future, with factors, such as dsRNA base sequences, sequence length, number of phosphate groups at the 5’-terminus and base modifications taken into comprehensive consideration in the reference standard preparation process.

Safety: Organisms contain an abundance of circRNAs that play important roles in regulating gene transcription, expression, and cellular signal transduction pathways, with certain circRNAs capable of protein expression (80, 121). Whether exogenous circRNAs cause disruptions to the biological functions of naturally occurring circRNAs in the body is unknown. Residual linear RNA precursor molecules may cause stronger adverse reactions due to activating the excessive innate immune response. circRNA vaccines contain residual dsRNA content. As the occurrence of myocarditis after vaccination with mRNA vaccines has been reported to be associated with dsRNA residual in the vaccines (122), further research is required to ascertain whether circRNA vaccines may cause myocarditis.

## Author contributions

YB and ZL conceived the framework and main text of this review article. YB and DL wrote the draft. QM and ZL reviewed the manuscript. QH and JL searched the literatures. All authors contributed to the article and approved the submitted version.
