# Supplementary material for: Research progress on circular RNA vaccines
Source: Front Immunol. 2023 Jan 12;13:1091797. doi: 10.3389/fimmu.2022.1091797 (PMC9878156; doi:10.3389/fimmu.2022.1091797)
Supplement: Supplementary file 1 [file Table_1.docx]

Supplementary Material

Table 1. Quality control parameters and methods for circRNA vaccines at different process stages

| **Process stage** | **Quality control parameter** | **Method** |
| --- | --- | --- |
| DNA template | Content | High-performance liquid chromatography (HPLC) |
|  | Purity | HPLC |
|  | Sequence integrity | Electrophoresis |
|  | Sequence accuracy | Next-generation sequencing (NGS) or Sanger sequencing |
|  | Linearization efficiency | Ion exchange chromatography |
|  | Residual host cell DNA | Quantitative polymerase chain reaction (qPCR) |
|  | Residual host cell RNA | qPCR |
|  | Residual host cell protein | Enzyme-linked immunosorbent assay (ELISA) |
|  | Endotoxins | Limulus amebocyte lysate (LAL) assay |
|  | Microbial limit | Membrane filtration method |
| Purified circRNA bulk | Circularization rate | Capillary electrophoresis (CE)/ HPLC |
|  | T4 RNA ligase (if present) | ELISA |
|  | Ribonuclease R (RNase R) (if present) | ELISA |
|  | Product-related impurities (linear RNA precursors and deciduous RNA) | CE/ HLPC |
|  | CircRNA sequence accuracy | Sequencing |
|  | CircRNA purity | CE/ HPLC |
|  | dsRNA | ELISA/dot-blot hybridization |
|  | CircRNA sequence integrity | Electrophoresis |
|  | RNA concentration and purity | Ultraviolet (UV) spectroscopy |
|  | In vitro residual transcriptase | ELISA |
|  | Residual DNA template | qPCR |
|  | Residual solvent | Gas chromatography-mass spectrometry (GC-MS) |
|  | Turbidity | Turbidity meter |
|  | pH | pH meter |
|  | In vitro antigen expression | ELISA after cell transfection |
|  | Endotoxins | LAL assay |
|  | Microbial limit | Membrane filtration method |
| Final bulk | Confirmation of circRNA sequences | Sequencing |
|  | circRNA purity | CE/ HPLC |
|  | Delivery system components | HPLC |
|  | Encapsulation efficiency | Ribogreen assay |
|  | Particle size and dispersion coefficient | Dynamic light scattering |
|  | Zeta potential | Dynamic light scattering |
|  | pH | pH meter |
|  | Neutralizing antibody level | Live virus neutralization assay |
|  | Cellular immune response | Enzyme-linked immunosorbent spot (ELISpot)/flow cytometry |
|  | In vitro antigen expression | ELISA after cell transfection |
|  | Endotoxins | Tachypleus amebocyte lysate (TLA) assay |
|  | Microbial limit | Membrane filtration method |
| Final Lot | circRNA purity | CE/ HPLC |
|  | circRNA identification | Sequencing |
|  | Particle size and dispersion coefficient | Dynamic light scattering |
|  | Zeta potential | Dynamic light scattering |
|  | pH | pH meter |
|  | Encapsulation efficiency | Ribogreen assay |
|  | Lipid content | HPLC |
|  | Excipient content | HPLC |
|  | Physical appearance | Visual inspection |
|  | Visible foreign matter | Lamp inspection |
|  | Insoluble particles | Visual inspection |
|  | Loading/loading difference | Volumetric method |
|  | Osmotic pressure | Osmometer |
|  | Residual moisture | Karl Fischer titration |
|  | Residual solvent | Gas Chromatography-Mass Spectrometer |
|  | Lipid impurities | HPLC |
|  | Lipid degradation impurities | HPLC |
|  | Neutralizing antibody level | Live virus neutralization assay |
|  | Cellular immune response | ELISpot/flow cytometry |
|  | In vitro antigen expression | ELISA after cell transfection |
|  | Endotoxins | TLA assay |
|  | Microbial limit | Membrane filtration method |
|  | Abnormal toxicity | Animal testing |

Note: Green font denotes quality control items that are also used for linear mRNA vaccines, red font denotes newly established quality control items, and blue font denotes quality control items that require confirmation.
